# Identification of Long Non-Coding RNAs Deregulated in Multiple Myeloma Cells Resistant to Proteasome Inhibitors

**DOI:** 10.3390/genes7100084

**Published:** 2016-10-06

**Authors:** Ehsan Malek, Byung-Gyu Kim, James J. Driscoll

**Affiliations:** 1 Division of Hematology and Oncology, University Hospitals, Case Medical Center, Seidman Cancer Center, Cleveland, OH 44106, USA; ehsan.malek@uhhospitals.org; 2 Department of Pediatrics, Case Comprehensive Cancer Center, Case Western Reserve University, Cleveland, OH 44106, USA; bxk93@case.edu; 3 Division of Hematology and Oncology, University of Cincinnati College of Medicine, Cincinnati, OH 45267, USA; 4 The Vontz Center for Molecular Studies, University of Cincinnati College of Medicine, Cincinnati, OH 45267, USA

**Keywords:** long non-coding RNA, multiple myeloma, myelomagenesis, proteasome, drug resistance

## Abstract

While the clinical benefit of proteasome inhibitors (PIs) for multiple myeloma (MM) treatment remains unchallenged, dose-limiting toxicities and the inevitable emergence of drug resistance limit their long-term utility. Disease eradication is compromised by drug resistance that is either present de novo or therapy-induced, which accounts for the majority of tumor relapses and MM-related deaths. Non-coding RNAs (ncRNAs) are a broad class of RNA molecules, including long non-coding RNAs (lncRNAs), that do not encode proteins but play a major role in regulating the fundamental cellular processes that control cancer initiation, metastasis, and therapeutic resistance. While lncRNAs have recently attracted significant attention as therapeutic targets to potentially improve cancer treatment, identification of lncRNAs that are deregulated in cells resistant to PIs has not been previously addressed. We have modeled drug resistance by generating three MM cell lines with acquired resistance to either bortezomib, carfilzomib, or ixazomib. Genome-wide profiling identified lncRNAs that were significantly deregulated in all three PI-resistant cell lines relative to the drug-sensitive parental cell line. Strikingly, certain lncRNAs deregulated in the three PI-resistant cell lines were also deregulated in MM plasma cells isolated from newly diagnosed patients compared to healthy plasma cells. Taken together, these preliminary studies strongly suggest that lncRNAs represent potential therapeutic targets to prevent or overcome drug resistance. More investigations are ongoing to expand these initial studies in a greater number of MM patients to better define lncRNAs signatures that contribute to PI resistance in MM.

## 1. Introduction

Multiple myeloma (MM) is characterized by the clonal proliferation of malignant plasma cells (PCs) within the bone marrow (BM) microenvironment [1,2]. MM is the second most common hematologic malignancy, with an incidence of 24,000 cases per year in the USA, and accounts for 2% of deaths from all cancers and ≈20% of deaths from hematologic cancers [3]. In Western countries, in the near future both the incidence and prevalence of this disease will increase as the result of an aging population and increased survival of those with the disease. As the number of patients surviving with MM increases, concern for the development of therapeutic resistance rises. Although the introduction of immunomodulatory drugs (IMiDs) and proteasome inhibitors (PIs) has improved the outlook, MM remains incurable. Induction therapy followed by autologous BM transplant forms the backbone of treatment for young and transplant-eligible patients. However, many patients do not respond to standard therapy and those that do respond inevitably develop resistance [4].

Eukaryotic cells maintain a healthy proteome through integration of the ubiquitin (Ub) + proteasome system (UPS) and aggresome + autophagy pathway [5]. The UPS plays a pivotal role in maintaining proteostasis through the selective elimination of misfolded, damaged, and short-lived proteins [6,7,8]. Molecules that inhibit the proteasome and disrupt proteostasis are selectively cytotoxic to cancer cells and have been exploited for therapeutic gain. Functional blockade of the UPS represents a remarkable bench-to-bedside success that has catapulted the proteasome into a position of prominence in cancer biology [9,10,11,12].

PIs such as bortezomib (Velcade™, Takeda Oncology, Cambridge, MA, USA) and carfilzomib (Kyprolis^®^, Amgen, Thousand Oaks, CA, USA) have improved the quality of life (QOL) and overall survival (OS) of MM patients. Bortezomib is a selective drug that inhibits the proteasome to exploit its pivotal cellular function and promotes tumor cell death. Ixazomib (Ninlaro^®^, Millennium, Takeda Oncology, Cambridge, MA, USA) is a boron-based, orally available PI, and was recently Food and Drug Administration (FDA) approved [13,14,15,16]. PIs also induce aggresome formation and autophagy as compensatory protein clearance mechanisms and this can lead to the emergence of drug resistance and disease relapse, leading to treatment failure and fatal outcomes. While the therapeutic benefit of targeting the proteasome remains unchallenged, more precise modalities that do not induce the aggresome + autophagosome pathway are needed.

Only one-fifth of transcription leads to protein-coding genes across the human genome, indicating that there are four times as many non-coding RNAs (ncRNAs) compared to coding RNAs. Long non-coding RNAs (lncRNAs) function as master regulators of the human genome and control the majority of key intracellular signaling pathways and processes including development, proliferation, differentiation, and apoptosis [17,18,19]. Accordingly, alterations in ncRNA expression are seen in tissues and contribute to cancers, autoimmune and genetic disorders, and infectious processes [20,21,22,23,24]. Specifically, deregulation of ncRNA levels is linked to tumor initiation, metastasis, and drug resistance. Thus, ncRNAs have rapidly attracted attention as potential diagnostic and therapeutic targets. lncRNAs are transcripts longer than 200 nucleotides. This arbitrary limit differentiates this group from smaller regulatory RNA such as microRNAs (miRNAs), small nucleolar RNAs (snoRNAs), Piwi-interacting RNAs (piRNAs), and short interfering RNAs (siRNAs). Certain ncRNAs may represent new therapeutic targets to overcome drug resistance in cancer [25,26,27].

MM is characterized by genetic heterogeneity in terms of changes in gene expression compared to normal BM plasma cells [28,29,30]. These expression changes can be driven directly or indirectly by changes in signaling, e.g., due to altered external stimuli mediated by a changing microenvironment such as by miRNAs [31]. miRNAs are non-protein-coding RNAs that function as regulators of mRNA stability and translation. miRNAs act post-transcriptionally to repress the expression of their target genes, while an upregulation of gene expression has been found under specific conditions, e.g., with specific transcripts, in distinct cell types [32]. A single miRNA is typically involved in the regulation of several hundred mRNAs. In turn, several miRNAs regulate one cognate mRNA. Thus, miRNAs function in both physiological and pathological processes, e.g., differentiation, angiogenesis, apoptosis, development of cancer, metastasis, and drug resistance. Individual miRNAs and miRNA signatures have been previously identified in monoclonal gammopathy of unknown significance (MGUS) and MM and have been described as diagnostic or prognostic biomarkers and therapeutic targets [33,34,35,36,37,38,39,40,41,42,43,44,45,46,47,48]. Global miRNA profiling studies with impact on gene expression, biological relevance, and survival have been reported, and imply a possible association with MM pathogenesis and molecular subgroups in terms of specific chromosomal aberrations or gene expression-based high-risk groups. In addition, circulating and serum-derived miRNAs have also been identified in MM patients [49,50,51,52]. In contrast, lncRNAs have been less well studied [53,54,55,56]. In the present study, we identified miRNAs and lncRNAs significantly deregulated in MM cells resistant to the three FDA-approved PIs, bortezomib, ixazomib, and carfilzomib.

## 2. Materials and Methods

### 2.1. Cell Lines and Reagents

MM cell lines (MMCLs) were obtained from the National Cancer Institute, Bethesda, MD, USA and cultured in complete Roswell Park Memorial Institute (RPMI) media supplemented with 10% fetal calf serum and penicillin-streptomycin. Bortezomib, carfilzomib, and ixazomib were from ActiveBiochem (Maplewood, NJ, USA). All other chemicals used were reagent grade (Sigma Chemical, St. Louis, MO, USA).

### 2.2. Generation of PI-Resistant Cells

RPMI8226 cells were exposed to successively increased concentrations (3 nM, 5 nM, 10 nM, 20 nM, 50 nM, and 100 nM) of bortezomib, ixazomib, or carfilzomib to generate resistant cells. Parental cells were cultured under the same algorithm in vehicle (0.05% dimethyl sulfoxide, DMSO) alone.

### 2.3. Cell Growth and Proliferation

The effect of proteasome inhibitors on myeloma growth and proliferation was assessed by measuring XTT (Sigma) dye absorbance. First, 5 × 10^4^ cells were plated in 96-well plates and incubated in media that lacked phenol red. Cells were then treated with drugs at the indicated concentration (10 nM) and incubated for 72 h. XTT-phenazine methosulfate (PMS) mixture (50 μL, 1 mg/mL XTT; 20 μM PMS) was prepared and added to plates that were then incubated for 4 h. Absorbance was then measured using a BMG Labtech FLUOstar OPTIMA plate reader (Ortenberg, Germany).

### 2.4. Detection of Apoptosis

First, 1 × 10^6^ myeloma cells were cultured for 24 h in a medium with or without PIs. Cells were harvested, washed, and stained with annexin V/propidium iodide (PI). Annexin V^+^/PI^−^ apoptotic cells were enumerated using the Epics flow cytometer (Beckman Coulter, Indianapolis, IN, USA). The percentage of cells undergoing apoptosis was defined as the sum of early apoptosis (annexin V^+^) and late apoptosis (annexin V^+^ and PI^+^) cells. Annexin V fluorescein isothiocyanate (FITC) conjugate was added and the sample was analyzed using a Coulter^®^ epics^®^ XL-MCL system (Beckman Coulter, Indianapolis, IN, USA).

### 2.5. Gene Expression Microarray

Total RNA that contained both mRNA and ncRNA was isolated from patient samples using the RNeasy kit (Qiagen Inc., Germantown, MD, USA). The quality of the total RNA was confirmed using the Agilent 2100 Bioanalyzer (Agilent, Santa Clara, CA, USA) and the RNA6000 Nano assay (Agilent). For each sample, the 3′ in vitro translation (IVT) express kit (Affymetrix, Santa Clara, CA, USA) synthesized biotin-labeled RNA target from 100 ng of the total RNA sample. The 3′ IVT kit contains hybrid primers that bind polyA (polydT) as well as random hexamer primers that bind ncRNA sequence. The kit generates cDNA to both polyA (coding) and non-polyA (non-coding) RNAs. A hybridization cocktail that included 10 μg of target was created for each sample. Samples were hybridized to the Genechip Primeview Human Gene Expression probe array cartridge (Affymetrix). The PrimeView Human Gene Expression Array provides comprehensive coverage of the human genome in a cartridge array format. The array is comprised of >530,000 probes covering >36,000 transcripts and variants, which represent >20,000 genes. Arrays contain probes for >20,000 total mature miRNAs, snoRNAs, ncRNAs, and pre-miRNAs. Those probes that demonstrated a cutoff greater or less than 2-fold from normal PCs were further analyzed.

### 2.6. Biostatistical Analysis

ncRNA profiles from drug-naïve and drug-resistant myeloma cells were performed and the statistical significance of the differences was determined using the Student *t* test with a minimal level of significance of *p* < 0.05.

## 3. Results

### 3.1. Generation of Myeloma Cells Resistant to Proteasome Inhibitors

**Figure 1:**
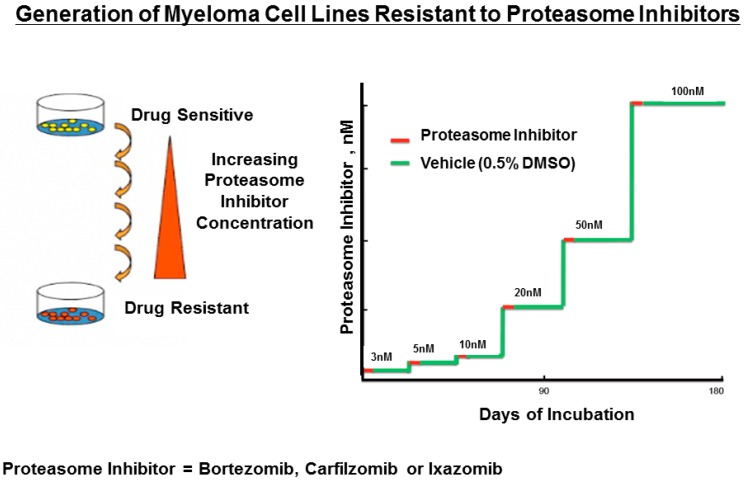
Scheme to generate myeloma cell lines resistant to proteasome inhibitors. Drug-naïve parental RPMI8226 cells were exposed to either vehicle (dimethyl sulfoxide (DMSO) 0.05%) or bortezomib, carfilzomib, or ixazomib at indicated concentrations. Cells were exposed to the vehicle or drugs for three days, pelleted, washed, grown in fresh media for three weeks, and then exposed to the vehicle or drug at the higher concentration.

**Figure 2:**
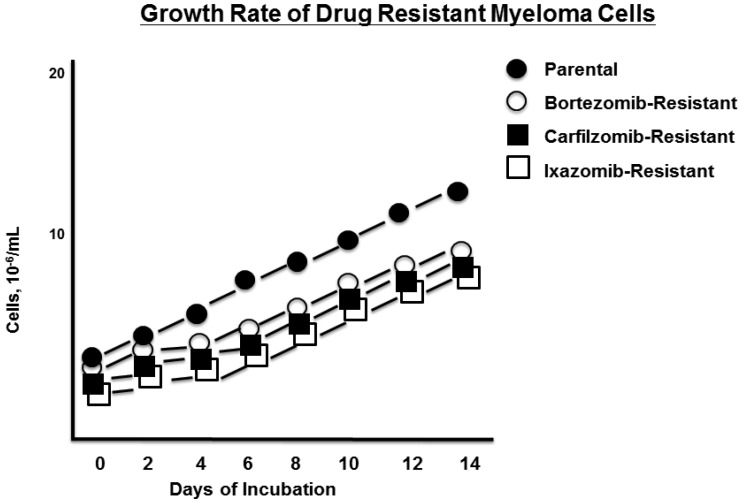
Growth rate of drug-resistant myeloma cells. The growth rate of parental and drug-resistant cells was determined by counting live cells by trypan blue staining. Shown is the average of triplicate measurements.

RPMI8226 myeloma cells were treated with either vehicle (0.05% DMSO) or the proteasome inhibitors (PIs) bortezomib, carfilzomib, or ixazomib (Figure 1). Over a period of six months, RPMI8226 cells were exposed to the PIs at successively increased concentrations that ranged from 1 nM up to 100 nM. Each of the three drug-resistant cell lines exhibited a reduced growth rate, as shown by trypan blue staining relative to the drug-naïve parental RPMI8226 cells (Figure 2).

### 3.2. Drug-Resistant Cells Are Less Sensitive to Proteasome Inhibitor Effects on Cell Viability and Apoptosis

**Figure 3:**
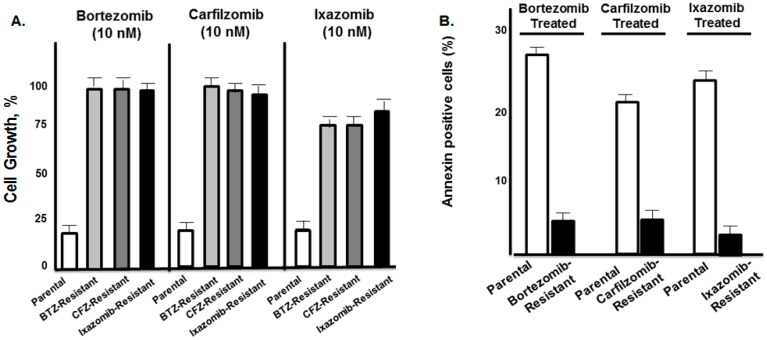
Effect of proteasome inhibitors (PIs) on drug-resistant multiple myeloma (MM) cell lines. (**A**) Effect of PIs on parental and drug-resistant cell viability. Parental, bortezomib (BTZ)-, carfilzomib (CFZ)-, or ixazomib (IXZ)-resistant cells were exposed to bortezomib, carfilzomib, or ixazomib (10 nM). The XTT assay was used to measure the effect of drugs on MM growth and proliferation. (**B**) Effect of PIs on the induction of apoptosis in the parental (drug-sensitive) and drug-resistant RPMI8226 cells. Cells were exposed to each PI (10 nM) for 18 h and the percentage of annexin-positive cells was determined by flow cytometry. Shown is the average of triplicate measurements.

Parental and PI-resistant cells were treated with bortezomib, carfilzomib, or ixazomib and the effect on cell growth and proliferation was determined using the XTT assay (Figure 3A). Importantly, each drug-resistant cell line was also resistant to the other two PIs, while the parental cells were sensitive to all PIs. The viability of drug-resistant cells was not affected by the PIs (10 nM), while the growth of parental cells was reduced by ≈80%. The PIs also induced apoptosis in parental cells, as determined by flow cytometry to detect annexin-positive cells (Figure 3B). We determined that ≈22%–28% of parental cells were annexin-positive after treatment with the three PIs but only 4%–6% of the drug-resistant cells were annexin-positive (Figure 3B).

### 3.3. Genome-Wide ncRNA Profiling of Parental and Drug-Resistant Myeloma Cells

**Figure 4:**
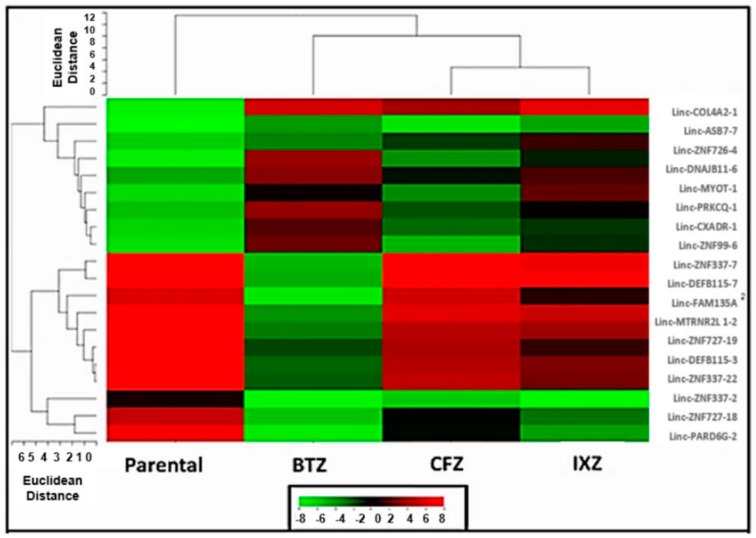
Heat map of long non-coding RNAs (lncRNAs) deregulated in bortezomib (BTZ)-, carfilzomib (CFZ)-, and ixazomib (IXZ)-resistant cells relative to parental cells. Shown are the lncRNAs significantly up- or downregulated in each PI-resistant cell line relative to drug-naïve parental cells. Genome-wide non-coding RNA (ncRNA) expression profiles from drug-resistant cells were compared to those of drug-naïve parental cells treated with vehicle alone (0.05% DMSO).

To detect ncRNAs and lncRNAs that correlated with PI resistance, total RNA was isolated from parental and the three PI-resistant cell lines. ncRNA from these cells was then analyzed using a global, unsupervised approach with Affymetrix-based microarrays. While over 15,000 pre-miRNAs and mature ncRNAs were screened, only 18 human ncRNAs (8 miRNAs and 10 lncRNAs) were expressed with statistically significant differences between the drug-naïve parental cells and the drug-resistant cell lines. Table 1 lists individual miRNAs that were significantly deregulated in PI-resistant cell lines relative to the parental PI-sensitive RPMI8226 cell line. Interestingly, miRNAs 29a, 29b, 29c and the Let-7 family have been previously linked to tumorigenesis [57,58,59]. As shown in the heat map (Figure 4) and Table 2, lnc-Col4A2-1, lnc-PRKCQ-1, lnc-DNAJB11-6, and lnc-MYOT-1 were significantly upregulated in all three PI-resistant cell lines. lncRNAs commonly downregulated in all three PI-resistant cells were also detected. These were lnc-PARD6G-2, lnc-FAM135A, lnc-MTRNR2L1-2, lnc-CD99-6, lnc-CHM-1, lnc-ZNF337-7, and lnc-CXorf64-1 (Figure 4).

### 3.4. Genome-Wide lncRNA Profiling of Healthy and MM Plasma Cells

**Figure 5:**
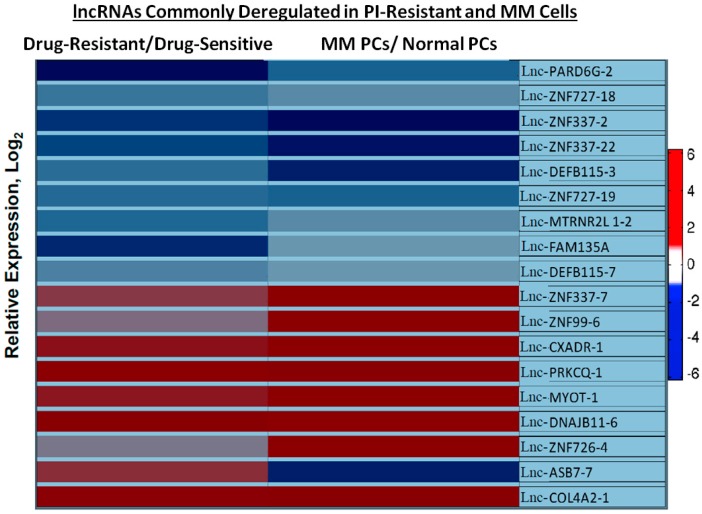
Comparison of lncRNAs deregulated in both drug-resistant cells relative to drug-sensitive cells and MM patient plasma cells (PC) relative to healthy plasma cells. lncRNAs commonly deregulated between the three PI-resistant cell lines with drug-sensitive parental RPMI8226 cells and in MM patient CD138+ cells relative to healthy CD138^+^ cells.

Preliminary studies were performed to identify individual lncRNAs that were deregulated in MM. Total RNA was isolated from CD138^+^ cells obtained from three healthy individuals and three newly diagnosed MM patients. lncRNA expression levels were determined using an unsupervised approach with Affymetrix-based microarrays that contained ≈11,000 lncRNA probes. The analysis identified eight different lnc-RNAs (lnc-Col4A2-1, lnc-ZNF726-4, lnc-DNAJB11-6, lnc-MYOT-1, lnc-PRKCQ-1, lnc-CXADR-1, lnc-ZNF99-6, and lnc-ZNF337-7) that were significantly upregulated in MM CD138^+^ cells compared to healthy CD138^+^ cells (Figure 5). Another 10 lnc-RNAs (lnc-ASB7-7, lnc-DEFB115-7, lnc-FAM135A, lnc-MTRN2L1-2, lnc-ZNF727-19, lnc-DEFB115-3, lnc-ZNF337-22, lnc-ZNF337-2, lnc-ZNF727-18, and lnc-PARD6G-2) were significantly downregulated in MM CD138^+^ cells compared to healthy CD138^+^ cells (Figure 5). Then, we analyzed lncRNAs deregulated in PI-resistant cells relative to PI-sensitive cells. The results indicated that lnc-Col4A2-1, lnc-ZNF726-4, lnc-DNAJB11-6, lnc-MYOT-1, lnc-PRKCQ-1, lnc-CXADR-1, and lnc-ZNF99-6 were upregulated in PI-resistant cells relative to parental cells. These same lncRNAs were also upregulated in MM CD138^+^ cells relative to healthy CD138^+^ cells. Figure 5 lists those lncRNAs deregulated in both PI-resistant cells and MM CD138^+^ cells.

## 4. Discussion

The identification of lncRNAs deregulated in myeloma cells resistant to PIs provides exciting new information to help unravel drug resistance mechanisms and identifies potential new therapeutic targets to improve cancer treatment. In healthy cells under physiologic conditions, multiple lncRNAs converge to maintain a proper balance of proliferation, differentiation, and death (Table 3). In pathologies such as human cancers, lncRNAs are deregulated and have profound consequences on viability and therapeutic response. Importantly, a single lncRNA can regulate multiple targets, e.g., miRNAs, mRNAs, and genes. In cancer, the loss of a tumor-suppressive lncRNA may enhance the expression of target oncogenes, whereas increased expression of an oncogenic lncRNA may repress tumor suppressors. This realization has resulted in studies to understand the pathways regulated by lncRNAs using cell-based pre-clinical model systems and to comprehend the feasibility of restoring tumor-suppressive lncRNAs for cancer therapy.

A recent report investigated lncRNAs as biomarkers for predicting survival in MM patients (55). In addition, metastasis-associated lung adenocarcinoma transcript 1 (MALAT1) has been found to be overexpressed in MM and may represent a marker to predict MM progression (53). Finally, a recent study identified deregulated lncRNAs putatively associated with MM pathogenesis and possibly linked to the accepted multi-step process underlining the different stages of the disease (59). The present study was focused on the uniformly fatal plasma cell dyscrasia MM as a model to study the role of lncRNAs in cancer and drug resistance. Through such an approach, microarray-based profiling revealed a panel of lncRNAs differentially regulated in myeloma cells. The molecular basis of resistance to proteasome inhibitors is a fundamental biologic problem that impedes translational capacity and therapeutic efficacy. Through correlative clinical and cytogenetic study of this disease, it is clear that what is called MM is a constellation of clinico-pathological findings characterized by genomic abnormalities, molecular pathogenesis, and clinical behavior. Ongoing studies are determining the role of individual ncRNAs and lncRNAs on protein ubiquitination pathways and the catalytic activity of the proteasome. However, currently there is no evidence that links lncRNAs to the UPS and provides a mechanistic link to drug resistance.

To the best of our knowledge, this is the first report describing the global expression profiles of lncRNAs in samples of MM from patients and PI-resistant MM cells. Notably, our study identified 8 lncRNAs that were specifically upregulated in both MM CD138^+^ cells compared to healthy CD138^+^ cells and PI-resistant cells relative to parental cells. Another nine lncRNAs were significantly downregulated in both MM CD138^+^ cells compared to healthy CD138^+^ cells and PI-resistant cells relative to parental cells (Figure 5). Despite the fact that the mechanism and the function of lncRNAs, as well as the consequences of their deregulation, remain to be fully clarified, many of the individual lncRNAs identified here that are deregulated in MM CD138^+^ cells are also deregulated in PI-resistant MM cells. Therefore, PI-resistant MM cell lines represent a reasonable model system to investigate the role of individual lncRNAs in the disease process and drug-resistance. Many of the prior studies that investigated ncRNAs in MM, have not looked at individual patients but rather present the collective average of up- and downregulated ncRNAs in all patients.

**Figure 6:**
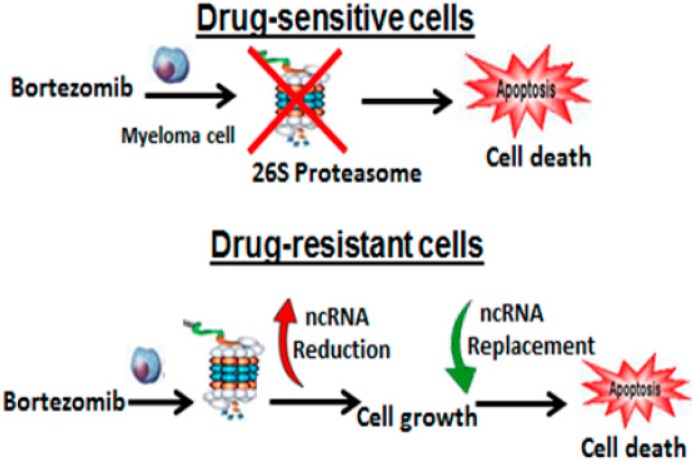
Model for lncRNA replacement therapy in MM. Bortezomib inhibits the proteasome in drug-sensitive MM cells leading to cell death. lncRNAs are reduced in drug-resistant myeloma cells leading to drug-resistance. Replacement of synthetically-engineered lncRNAs restores sensitivity to proteasome inhibitors and overcomes drug resistance.

ncRNA replacement therapy is an innovative strategy that could save patients from ineffective treatment, improve QOL, enhance OS, and reduce the devastating impact of cancer therapy on the healthcare system [55,56,57,58] (Figure 6). Certain ncRNAs function as bona fide tumor suppressors and synthetic versions of these ncRNAs robustly interfere with tumor growth in animal models to strongly support the investigation and clinically development of ncRNA replacements. There are two main therapeutic strategies related to targeting ncRNAs. ncRNAs that acquire a gain-of-function in the diseased tissue, e.g., oncogenic ncRNAs, can be inhibited by using ncRNA antagonists, such as antagomirs. Alternatively, ncRNAs that show a loss of function, e.g., tumor suppressors, can be restored by using ncRNA mimics or replacements. ncRNA replacement therapy involves the re-introduction of ncRNAs into diseased tissues to reactivate pathways that drive a therapeutic response. Reactivation of these ncRNA-regulated pathways can interfere with the oncogenic properties of cancer cells, blocking uncontrolled proliferation and inducing apoptosis. Based upon our pre-clinical studies, we propose to investigate these ncRNAs as attractive targets for therapeutic intervention. Ultimately, our goal is to understand the roles of ncRNAs in cancer and the potential for manipulating miRNAs for cancer therapy as these molecules make their way towards the clinic.

Theragnostics (a portmanteau of therapeutics and diagnostics) incorporate multiple disciplines, e.g., bioinformatics, pharmacogenomics, proteomics, and metabolomics, to design accurate diagnostic assays with a targeted therapy that is selected based upon testing results [62,63]. The strategy offers for an advanced molecular understanding of cancer, to develop more effective molecular targets and to design therapeutic agents based upon patient-specific biology of disease. As a result, patients should receive personalized, targeted therapy tailored to the specific biological and molecular features of their tumor. Accurate diagnostic testing is also designed to avoid the unnecessary treatment of patients that are not likely to demonstrate a significant clinical response or for whom a specific therapy is not appropriate. The wide-scale use of theragnostics requires further bioinformatic and genomic advances as standardized tools in predictive medicine. These are key to the development and implementation of personalized therapies that eventually will improve the OS of cancer patients. In oncology, the approach is aimed at a more accurate diagnosis of cancer and optimization of patient selection to identify those most likely to overcome drug resistance. ncRNAs are readily detected in body fluids, for example, serum, plasma or urine, as well as circulating tumor cells to demonstrate their potential as readily accessible, non-invasive diagnostic and prognostic biomarkers and potential therapeutics. Specific ncRNAs are aberrantly expressed early in myelomagenesis and may therefore more readily detect high-risk disease than current methods. Although only recently discovered, ncRNAs have rapidly advanced from preclinical studies to evaluation in human clinical trials. The development of ncRNA theragnostics should provide widely applicable tools for the targeted delivery of personalized medicines to improve the outcome of patients with MM.

## 5. Conclusions

We have identified a curated panel of deregulated lncRNAs in common within myeloma cells generated with acquired resistant to three different clinically-relevant PIs and MM patients. Our study has shown the value of PI-resistant cell lines as a preliminary model system to address the mechanistic role of individual lncRNAs in MM and the development of drug resistance. The results prioritize specific lncRNAs to be studied in terms of defining their role in myelomagenesis and the development of drug resistance. Further investigation is warranted to shed light on the precise mechanism of action of these lncRNAs and identify their biological functions and targets.

## Figures and Tables

**Table 1:** MicroRNAs (miRNAs) down- or upregulated in the bortezomib-, carfilzomib-, and ixazomib-resistant cells compared to parental RPMI8226 cells. (**A**) Individual miRNAs that were downregulated at least 10-fold in all three of the proteasome inhibitor (PI)-resistant RPMI8226 cell lines compared to parental RPMI8226 cells. (**B**) Individual miRNAs that were upregulated at least 10-fold in all three of the PI-resistant RPMI8226 cell lines compared to parental RPMI8226 cells.

A. Downregulated miRNAs	B. Upregulated miRNAs
29a	Let-7A2
29b	Let-7D
29c	Let-7E
Let-7A1	Let-7F2

**Table:** **A.**

LncRNA ID	BTZ/Parental		CFZ/Parental		IXZ/Parental		Chromosome Location	Start Sequence	Stop Sequence
	Fold	Absolute	Fold	Absolute	Fold	Absolute			
PARD6G-2	−34	−2814	−6.9	−2482	−9.4	−2591	18p1	106602	108335
FAM135A	−21	−1516	−2.0	−818	−3.5	−1139	6q13	71104590	71104590
MTRNR2L12	−19	−687	−1.5	−253	−4.1	−549	17p11	21516455	21548969
CD99-6	−9.6	−714	−1.5	−275	−2.5	−545	X	1862407	18627778
CHM-1	−8.5	−418	−1.6	−193	−3.8	−352	X	86979061	86983512
ZNF337-1	−7.2	−365	−3.1	−287	−4.9	−338	20p11	26113523	26114834
CXorf64-1	−5.4	−188	−2.2	−127	−3.7	−168	X	125243745	125249545

**Table:** **B**

LncRNA ID	BTZ/Parental		CFZ/Parental		IXZ/Parental		Chromosome Location	Start Sequence	Stop Sequence
	Fold	Absolute	Fold	Absolute	Fold	Absolute			
COL4A2-1	16	447	12.8	346	28.1	797	13q34	11074616	110774945
SFMBT2	4.5	5072	2.7	2381	4.0	4200	10p14	7487520	7513904
MYOT-1	4.1	182	1.7	35	2.3	78	5q31	136070616	136090375
PRKCQ-1	4.5	156	NA	22	2.9	84	10p15	6933419	6977358
CHM-1	3.0	142	NA	25	2.3	73	4p16.1	6765264	6769333

**Table 3:** Shown are lncRNAs upregulated (green up arrow) or downregulated (red down arrow) in PI-resistant MM cells and MM tumor cells. Chromosomal location, strand type, and putative gene or ncRNA targets are also shown based upon searches using http://lncipedia.org/ and http://www.ncbi.nlm.nih.gov/ [60,61].

lncRNA	PI Resistant	MM Patients	Chromosome	Putative Target
lncRNA PARD6G-2	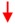	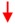	18p11(-)	PARD6G-AS1
lncRNA ZNF727-18	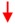	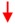	7q11(+)	Unknown
lncRNA ZNF337-2	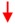	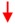	20p11(-)	ZNF337-AS-1
lncRNA ZNF337-22	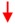	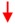	20p11(-)	Unknown
lncRNA DEFB115-3	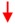	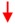	20q11(+)	Unknown
lncRNA ZNF727-19	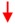	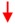	7q11(+)	Unknown
lncRNA MTRNR2L-12	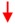	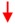	17p11(+)	lnc-MTRNR2L-12
lncRNA FAM135A	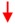	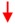	6q13(+)	miR-4436a
lncRNA DEFB115-7	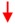	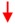	20q11(+)	Unknown
lncRNA ZNF337-7	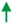	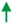	20p11(-)	Unknown
lncRNA ZNF99-6	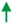	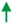	19p12(-)	Unknown
lncRNA CXADR-1	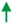	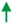	21q21(+)	Unknown
lncRNA PRKCQ-1	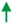	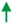	10p15(-)	miR-4295
lncRNA MYOT-1	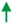	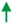	5q31(+)	Unknown
lncRNA DNAJB11-6	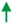	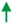	3q27(+)	Unknown
lncRNA ZNF726-4	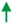	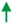	19p12(+)	Unknown
lncRNA ASB7-7	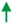	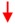	15q26(+)	Unknown
lncRNA COL4A2-1	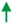	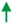	13q34(+)	miR-485-5p
